# Neutrophil Cytosolic Factor 1 in Dendritic Cells Promotes Autoreactive CD8^+^ T Cell Activation via Cross-Presentation in Type 1 Diabetes

**DOI:** 10.3389/fimmu.2019.00952

**Published:** 2019-05-01

**Authors:** Chao Liu, Robert L. Whitener, Andrea Lin, Yuan Xu, Jing Chen, Alexei Savinov, Jennifer W. Leiding, Mark A. Wallet, Clayton E. Mathews

**Affiliations:** ^1^Department of Pathology, Immunology and Laboratory Medicine, University of Florida Diabetes Institute, Gainesville, FL, United States; ^2^Department of Medicine, University of Florida, Gainesville, FL, United States; ^3^Children's Health Research Center, Sanford Research, Sioux Falls, SD, United States; ^4^Division of Allergy and Immunology, Department of Pediatrics, Johns Hopkins-All Children's Hospital, University of South Florida, St. Petersburg, FL, United States

**Keywords:** Reactive oxygen species, Ncf1, NADPH oxidase 2, type 1 diabetes, dendritic cell, cross-presentation, CD8+ T cell

## Abstract

**Aims:** Reactive oxygen species (ROS) are critical in driving the onset of type 1 diabetes (T1D). Ablation of ROS derived from phagocytic NADPH oxidase 2 is protective against autoimmune diabetes in non-obese diabetic (NOD*)* mice. However, the mechanisms of NADPH oxidase 2-derived ROS in T1D pathogenesis need to be elucidated. Here, we have examined the role of *Ncf1* (the regulatory subunit of NADPH oxidase 2) in dendritic cells (DC).

**Results:** *Ncf1*-mutant DCs exhibit reduced ability to activate autoreactive CD8^+^ T cells despite no difference in co-stimulatory molecule expression or pro-inflammatory cytokine production. When provided with exogenous whole-protein antigen, *Ncf1*-mutant NOD DCs showed strong phagosome acidification and rapid antigen degradation, which lead to an absence of protein translocation into the cytoplasm and deficient antigenic peptide loading on MHC Class I molecules.

**Innovation:** This study demonstrates that Ncf1 (p47^phox^) is required for activation and effector function of CD8^+^ T cells by acting both intrinsically within the T cell as well as within professional antigen presenting cells.

**Conclusion:** ROS promote CD8^+^ T cell activation by facilitating autoantigen cross-presentation by DCs. ROS scavengers could potentially represent an important component of therapies aiming to disrupt autoantigen presentation and activation of CD8^+^ T cells in individuals at-risk for developing T1D.

In Type 1 Diabetes (T1D), pancreatic β cells are attacked by a T cell mediated autoimmune response and lose their ability to produce insulin (1–3). While a number of immune cell subsets are involved throughout the development of T1D, cytotoxic CD8^+^ T cells (CTLs) function as primary effectors of β cell damage. To this end, CTL are the most abundant cell type within T1D patient islet infiltrates (4). In the T1D-prone non-obese diabetic (NOD) mouse, targeted deletion of CTLs or blocking CTL effector function prevents T1D development (5–9). To activate autoreactive naïve CTLs in T1D, dendritic cells (DC) must take up β cell derived autoantigens by phagocytosis and load autoantigen peptides onto MHC Class I molecules. During maturation, the DCs migrate to the pancreatic draining lymph nodes (PLN) where, in conjunction with help signals provided by antigen specific CD4^+^ T cells, they present the autoantigen peptides to CTLs, via a process termed cross-presentation (10–13). These activated CTLs then emigrate from the PLN and traffic to the islets where they recognize and kill β cells presenting autoantigens (2). In the NOD model, the process of cross-presentation is essential in the occurrence of spontaneous T1D as a deficiency in the cross-priming of CTLs prevents T1D onset (13).

The process of antigen cross-presentation begins after phagocytosis or pinocytosis. Within the phagosomes or endosomes, proteolytic enzymes quickly degrade whole proteins into smaller peptides suitable for presentation by MHC Class II. Antigen degradation in phagosomes or endosomes is under tight control, and under the appropriate conditions, intact protein can be exported into the cytoplasm and become involved in mechanisms for presentation by MHC Class I. There, the proteins are processed into small peptides by the proteasome/immunoproteasome and loaded onto MHC class I molecules in the ER or Ergosomes (14). Failure to regulate such pino/phagosomal antigen processing may interrupt cross-presentation by over-degrading the antigens. DCs regulate antigen degradation via multiple mechanisms, and reactive oxygen species (ROS) have been proposed as vital for effective DC cross-presentation (15–17).

ROS are a group of oxygen containing, highly reactive molecules (18) that exert numerous physical functions ranging from potent antimicrobial effects to signal transduction (18, 19). The NADPH oxidase family represents one of the major groups of ROS generating enzymes (19). NADPH oxidase 2 is widely expressed in immune cells (i.e., neutrophils, DCs, macrophages, and T cells) (20). During the course of an immune response, NADPH oxidase 2 within DCs has been proposed to attenuate antigen degradation and promote cross-presentation through production of ROS (15, 17, 21). However, two models of ROS in cross presentation show discrepancy regarding how ROS impact antigen degradation in cross presentation (15, 17). Previous studies have proposed that after phagocytosis, NADPH oxidase 2 is recruited to the phagosome and produces ROS (15, 22). These negatively charged free radicals can react with protons pumped in by the V-ATPase and thereby inhibit the phagosomal acidification (15), thus limiting the function of acid proteases, which require low pH for activity. However, Rybicka et al. concluded that ROS suppress antigen degradation in DC through a mechanism independent of pH regulation. In this second model, ROS inhibit local cysteine cathepsins through redox modulation (17). Elucidating the mechanism is essential to fully understanding the role of DC NADPH oxidase 2 during immune responses as well as in autoimmune disease such as T1D.

We have reported that NADPH oxidase 2 is indispensable for the development of T1D (23, 24). With a mutated and nonfunctional p47^phox^ subunit, NADPH oxidase 2 could not be activated to produce ROS, and CTL from p47^phox^ mutant NOD (NOD-*Ncf1*^*m*1*J*^) mice have compromised effector function (23). CTL with a deficient NADPH oxidase 2 were significantly less capable of transferring disease into NOD-*Scid* recipients, as compared with NOD CTLs (23). However, considering that NOD-*Ncf1*^*m1J*^ mice exhibit almost complete resistance against spontaneous T1D, the presence of T1D after transfer of NOD-*Ncf1*^*m1J*^ CTL provides rationale for further interrogation of this system to identify additional cell populations where NADPH oxidase two function could exert a pathogenic role.

In this study, we explored the capacity of DC isolated from NOD-*Ncf1*^*m1J*^ mice to activate autoreactive CD8^+^ T cells in the NOD mouse model of T1D. Here we show that NOD DC maturation, in terms of upregulation of surface stimulatory molecules and expression of pro-inflammatory cytokines, is not impacted by mutation of p47^phox^. However, cross-presentation of β cell antigens to autoreactive CD8^+^ T cells in NOD-*Ncf1*^*m1J*^ was deficient. In addition, our data support that NADPH oxidase 2 in the DC of NOD mice regulates antigen degradation through modulating phagosomal pH. These findings demonstrate for the first time the importance of Ncf1 in cross-presenting DC for activation of autoreactive CD8^+^ T cells and support the role of this enzyme in the pathology of autoimmune T1D.

## Materials and Methods

### Animals

NOD/ShiLtJ (NOD), NOD.Cg-*Rag1*^*tm*1*Mom*^ (NOD-*Rag1*^−/−^), NOD.Cg-*Rag1*^*tm*1*Mom*^Tg (TcraAI4)^1Dvs^/DvsJ (NOD.*AI4*α-*Rag1*^−/−^), and NOD.Cg-*Rag1*^*tm*1*Mom*^Tg (TcrbAI4)^1Dvs^/DvsJ (NOD.AI4β-*Rag1*^−/−^) mice were purchased from The Jackson Laboratory (Bar Harbor, ME). F1 hybrid progeny developed from outcrosses of NOD.AI4α-*Rag1*^−/−^ to NOD.AI4β-*Rag1*^−/−^ (hereafter referred to as NOD.AI4-*Rag1*^−/−^) developed diabetes between 3 and 5 weeks of age. NADPH oxidase 2 deficient NOD-*Ncf1*^*m1J*^ mice were generated as previously described (23, 24). NOD.*Ncf1*^*m1J*^.Cg-*Rag1*^*tm*1*Mom*^ (NOD-*Ncf1*-*Rag1*^−/−^) mice were generated through F2 mating of NOD-*Rag1*^−/−^ to NOD-*Ncf1*^*m1J*^. Mice were genotyped as described (25) to ensure homozygosity of *Rag1*^−/−^ as well as *D5Mit30* and *D5Mit31* to test for inheritance of the *Ncf1*^*m1J*^ allele. Mice at the F2 generation that were homozygous for the targeted deletion of *Rag1* and the mutant allele of *Ncf1* were used as founders for this mouse strain. Female mice were used for all experiments. All mice used in this study were housed in specific pathogen free facilities, and all studies herein were approved by the institutional animal care and use committee at the University of Florida.

### Materials

Fluorescently labeled antibodies including: Phycoerythrin (PE)-labeled α-CXCR4 (2B11), Brilliant violet 421-labeled α-CD8 (53-6.7), allophycocyanin (APC)-labeled α-CD3ε (BM8), and APC-labeled α-T-bet (4B10), PE-labeled α-granzyme B (NGZB), PE-labeled α-interferon gamma (IFNγ) (XMG1.2), APC labeled α-TNFα (MP6-XT22) [eBioscience (San Diego, CA)] as well as PE-labeled α-H2K^d^ [Biolegend (San Diego, CA)] were used. Recombinant mouse granulocyte-macrophage colony stimulating factor (rmGM-CSF) and rmIL-4 were purchased from R&D systems (Minneapolis, MN). Pam3CysSerLys4 (Pam3CSK4) and Polyinosinic-polycytidylic acid (Poly(I:C)) were purchased from Invivogen (San Diego, CA). Lipopolysachharide (LPS) was purchased from Sigma (St. Louis, MO). Polybead amino 3.0 μm microspheres were purchased from Polysciences (Warrington, PA). Horse cytochrome c was purchased from Sigma. Alexa Fluor 647 (AF647) and DQ ovalbumin (DQ-OVA) were purchased from Life technologies (Grand Island, NY). Fluorescein isothiocyanate (FITC) conjugated ovalbumin (OVA) was purchase from Sigma.

### Purification of T Cells

Mouse spleens or lymph nodes were collected, homogenized to a single cell suspension, and subjected to hemolysis with Gey's solution. Negative selection of CD8^+^ T cells from was performed using magnetic beads [mouse CD8^+^ T cell isolation kit (Miltenyi Biotec)], according to the manufacturer's protocol. CD4^+^ T cells from NOD as well as CD8^+^ T cells from NOD and NOD-*Ncf1*^*m1J*^ were purified by negative selection with magnetic beads according to the manufacturer's protocol using a CD4^+^ T cell isolation kit or a CD8^+^ T cell isolation kit (Miltenyi Biotec), respectively. Purity, >96%, was confirmed by flow cytometric analysis on a BD LSR Fortessa.

### Adoptive Transfer

Pre-diabetic (8 weeks old) NOD and NOD-*Ncf1*^*m1J*^ T cell donors were used for adoptive transfer experiments. Splenocytes were purified as described above. CD4^+^ and CD8^+^ T cells were mixed at a ratio of 3:1 and 10^7^ cells were transferred intraperitoneally (i.p.) to 8 week old NOD-*Rag1*^−/−^ or 8 week old NOD-*Ncf1*^*m1J*^*-Rag1*^−/−^ recipients. Transfers were divided into 4 groups: NOD-Rag1^−/-^ mice received either (1) NOD CD4^+^ + NOD CD8^+^ or (2) NOD CD4^+^ + NOD-*Ncf1*^*m1J*^ CD8^+^, while the remaining two groups were NOD-*Ncf1*^*m1J*^*-Rag1*^−/−^ recipients of (3) NOD CD4^+^ + NOD CD8^+^ or (4) NOD CD4^+^ + NOD-*Ncf1*^*m1J*^ CD8^+^. Mice were monitored weekly for diabetes onset as described previously (23). Engraftment of cells was confirmed by flow cytometry.

### Cell Culture

Bone marrow derived DCs (BMDCs) were generated by 8 days of culture in complete RPMI 1,640 media with 10% FBS (26). The culture media was supplemented with 1,000 U/mL rmGM-CSF and 500 U/mL rmIL4. Maturation was induced by 24-h treatment with 100 ng/mL Pam3CSK4, 25 μg/mL poly (I: C), 100ng/mL LPS, 1ug/mL R848, or 5 μg/mL CpG2336 respectively.

### Quantitative Real-Time Quantitative PCR

Real time quantitative PCR was performed as previously reported (27–31). In general, total RNA from DCs was isolated with TRIzol (Invitrogen, Carlsbad, CA) and cDNA was prepared using the Superscript III First-Strand Synthesis System (Invitrogen) according to the manufacturer's protocol. SYBR Green I (Bio-Rad) analysis was performed on a LightCycler 480 II (Roche, Basel, Switzerland). The amplification program utilized the following steps for all primer sets: 95°C for 10 min, then 45 cycles of 95, 60, and 72°C for 30 s. Melting-curves were performed for each PCR reaction to ensure specificity. Primers were used according to qPrimerDepot (NIH) and previous reports and listed as follows (28, 32):

**Table:** 

IFN-stimulated gene-15 (ISG-15)	forward, 5′-GAGCTAGAGCCTGCAGCAAT
	reverse, 5′-TAAGACCGTCCTGGAGCACT
IFN-regulatory factor-7 (IRF7)	forward, 5′-ACAGCACAGGGCGTTTTATC
	reverse, 5′-GAGCCCAGCATTTTCTCTTG
Mx1	forward, 5′-GATCCGACTTCACTTCCAGATGG
	reverse, 5′-CATCTCAGTGGTAGTCCAACCC
TNFα	forward, 5′-AGATGATCTGACTGCCTGGG
	reverse, 5′-CTGCTGCACTTTGGAGTGAT
IL12p35	forward, 5′-CTAGACAAGGGCATGCTGGT
	reverse, 5′-GCTTCTCCCACAGGAGGTTT
IL-10	forward, 5′-TGCTATGCTGCCTGCTCTTA,
	reverse 5'-TCATTTCCGATAAGGCTTGG.

### Flow Cytometry

Flow cytometry was performed to detect the surface proteins on and phagocytosis by BMDC. BMDC were counted and re-suspended in PBS at 2 × 10^7^ cells/mL. Approximately 10^6^ cells were labeled with antibodies at the proper dilution. Fluorescence was measured using a LSR Fortessa (BD Bioscience, San Jose, CA). Data were collected and analyzed using Flowjo 7.6.1 software.

### Measurement of Phagocytosis

Phagocytosis by BMDC was determined using beads coupled with the fluorescent pH insensitive indicator dye AF647. BMDC were incubated at 37°C for 20 min in the presence of fluorescent beads, washed extensively with cold PBS, and then immediately analyzed by flow cytometry. BMDCs with different numbers of bead phagocytized were gated by assessing fluorescence intensity of AF647. The percentages of cells that had taken up increasing numbers of beads were calculated based on the cell count of each gate to the total CD11c positive cells (**Figure 3**).

### Measurement of Phagosomal pH

Phagosomal pH was measured by fluorescence quenching of the pH sensitive dye Fluorescein 5-isothiocyanate (FITC) after phagocytosis (15, 22). A detailed experimental process was described by Savina et al. (22). Briefly, 3 μm Polybead® Amino Microspheres activated by 8% glutaraldehyde were incubated with Ovalbumin (OVA) conjugated with FITC (pH sensitive) and AF647 (pH insensitive) in PBS at 4°C overnight. After 30 min of aldehyde blocking with glycine (1M), labeled beads were washed three times and suspended in PBS at a concentration of 1.7 × 10^7^ beads per microliter. BMDCs were stained with α-CD11c antibody. After washing with PBS, BMDCs were incubated with the FITC/AF647 coupled beads for 15 min at 37°C, at a BMDC to bead ratio of 1:3 and washed three times with ice cold PBS to remove the excess beads. Bead pulsed BMDCs were re-suspended in warm RPMI media, incubated for the indicated time points. At each time point, BMDCs were immediately placed on ice and subjected to flow cytometry analysis. The mean fluorescence intensity (MFI) emission for both dyes was determined for BMDCs containing a single phagocytosed bead. The ratio of MFI between FITC and AF647 was employed to indicate the phaghosomal pH value. The phagosomal pH values were determined by establishing a standard curve. To develop the standard curve, BMDC were incubated with coupled beads for 30 min and then re-suspended in HBSS containing 0.1% Triton X-100 adjusted to a pH ranging from 5.5 to 8.0. After an 8-min incubation, these cells are immediately analyzed by flow cytometry and the emission ratio of the two fluorescent probes of BMDC incubated in HBSS at each pH was collected.

### Phagosomal Antigen Degradation Assay

Ovalbumin with a self-quenching conjugate that exhibits green fluorescence upon proteolytic degradation (DQ-OVA) and AF647 were covalently conjugated to 3 μm Polybead® Amino Microspheres as described above. Anti-CD11c labeled BMDC were pulsed with DQ-OVA/AF647 coupled beads for 15 min in 37°C media. Extracellular beads were washed away with cold PBS and BMDC were chased for a series of time periods. At each time point fluorescence was measured by flow cytometry and OVA degradation was calculated as the MFI ratio between DQ-OVA and AF647.

### Detection of Antigen Translocation

Horse cytochrome c was employed to measure antigen translocation from the phagosome into the cytoplasm for cross presentation as previously described (33). BMDC were seeded in 96-well plates and incubated with cytochrome c (0–4 mg/mL) for 24 or 48 h. BMDC apoptosis induced by cytoplasmic cytochrome c serves as an indicator of exogenous antigen being translocated into the cytoplasm (33, 34). BMDC not treated with cytochrome c were used as a negative control and cell viability assessed via MTT assay as previously reported (35).

### Measurement of T Cell Proliferation

Cell proliferation was measured by incorporation of radiolabeled tritiated thymidine ([^3^H]TdR). [^3^H]TdR was added to BMDC-T cell culture at 1 μCi per well for 16 h before harvesting. After harvest the radioactivity was measured as described previously (24).

### *In vitro* Cross Presentation Assays

The ability of BMDC to internalize whole protein antigen and then induce CD8^+^ T cell activation was assessed using an *in vitro* cross-presentation assay. To measure the cross-presentation of autoantigen, insulin reactive G9C8 and AI4 T cells were purified and incubated with BMDCs from NOD or NOD-*Ncf1*^*m1J*^ at a ratio of 5:1. Antigens (beads conjugated with Insulin, freeze thawed necrotic NIT-1 cells [a NOD derived β cell line (36)], heat-inactivated insulin as whole antigen, or insulin B15-23 peptide antigen) were added to the cell mixture. CD8^+^ T cell prolifeation was measured by [^3^H]TdR incorporation an assays (23)].

### Statistics

Statistical analysis was performed using GraphPad Prism (GraphPad Software, Inc., La Jolla, CA) or SAS 9.2 (SAS Institute Inc., Cary, NC). Statistical significance between mean values was determined using the Student's *t*-test with a significance level at *P* = 0.05. Time to diabetes onset after adoptive transfer was analyzed by Kaplan-Meier curve. The log-rank test was employed to compare the survival distributions of different groups and correct for multiple comparisons. Except for the survival analysis, all data reported here are representative of at least three independent experiments performed in triplicate.

## Results

### NOX2 Activity in Both Donor CD8+ T Cells and Endogenous Recipient Immune Cells Is Essential for Full Adoptive Transfer of T1D

**Figure 1:**
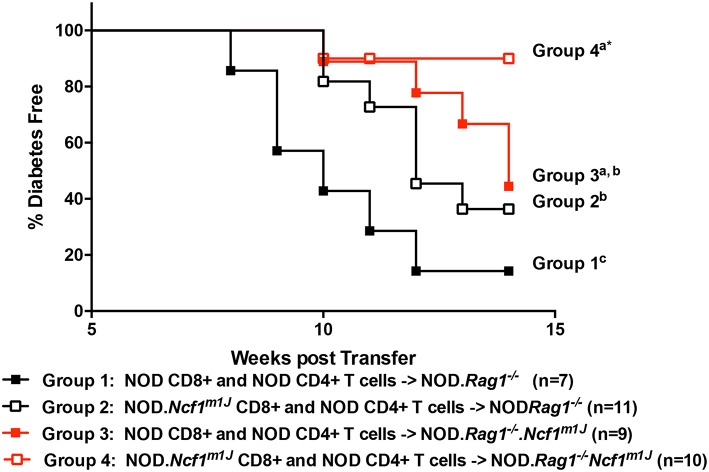
A functional Ncf1 is essential in both CD8^+^ T cells and the environment for full T1D pathogenesis. Purified NOD CD4^+^ T cells were co-transferred with purified CD8^+^ T cells from either NOD (closed symbols) or NOD.*Ncf1*^*m1J*^ (Open symbols) at a 3:1 ratio. All donors were 8-week-old prediabetic mice. A total of 10^7^ total T cells were injected i.p. into NOD.*Rag1*^−/−^ (black) or NOD.*Rag1*^−/−^.*Ncf1*^*m1J*^ (red) recipient female mice. Mice were monitored by glucosuria for the onset of diabetes and confirmed by blood glucose measurement. Mice were considered diabetic after two consecutive readings of blood glucose above 250 mg/dL. ^*^Letters denote significance: groups/lines with different letters were statistically significant, whereas those with the same letter were not statistically different. Table 1 contains *p*-values for the comparisons amongst groups.

To assess defects in the diabetogenicity of CTLs and antigen presenting cells (APCs) lacking intracellular superoxide production, purified naïve CD8^+^ T cells collected from prediabetic NOD or NOD-*Ncf1*^*m1J*^ donor mice were adoptively co-transferred with CD4^+^ T cells from prediabetic NOD into either NOD*-Rag1*^−/−^ or NOD*-Rag1*^−/−^*.Ncf1*^*m1J*^ recipient animals. Recipients were followed for onset of T1D. As previously noted (23), transfer of NOD.*Ncf1*^*m1J*^ CD8^+^ T cells into immune deficient NOD-*Rag1*^−/-^ mice resulted in a significant delay and reduction in disease onset when compared to NOD CD8^+^ T cells (Figure 1 and Table 1. Group 1 vs. Group 2: *p* = 0.05). Protection was also was also observed when transferring NADPH oxidase 2-competent immune cells (23) into NOD-*Rag1*^−/−^*.Ncf1*^*m1J*^ (Figure 1). Specifically, transfer of NOD CD4^+^ and CD8^+^ T cells into NOD-*Rag1*^−/−^*.Ncf1*^*m1J*^ recipients led to a reduction and delay in T1D onset compared to NOD*-Rag1*^−/−^ animals (Figure 1 and Table 1. Group 1 vs. Group 3: *p* = 0.03). Group 2 and Group 3 were statistically indistinguishable suggesting that cells within the NADPH oxidase 2 deficient recipient mice, likely DC, were providing protection against T1D similar to that observed with lack of NADPH oxidase 2 activity in CD8^+^ T cells (23). The group exhibiting the lowest frequency of diabetes development involved NOD-*Rag1*^−/−^*.Ncf1*^*m1J*^ recipients of NOD.*Ncf1*^*m1J*^ CD8^+^ T cells (Figure 1 and Table 1, Group 4). Indeed, groups 1 and 2 exhibited significantly more T1D when compared to Group 4 (Table 1). While 5 of 9 mice in Group 3 developed T1D compared to only 1 of 10 in Group 4 (Figure 1), statistical comparison of these two groups did not reach significance (Table 1: *p* = 0.09). Hence, there is a need for p47^phox^ within both CD8^+^ T cells and DCs for full T1D pathogenesis to be observed.

**Table 1:** Significance matrix for the adoptive transfer studies.

**Group #**	**Recipient strain**	**CD4 donor**	**CD8 donor**	***p*-value vs. group 1**	***p*-value vs. group 2**	***p*-value vs. group 3**	***p*-value vs. group 4**
1	NOD.*Rag1^−/−^*	NOD	NOD	^*^NA	^**§**^**0.05**	**0.03**	**0.01**
2	NOD.*Rag1^−/−^*	NOD	NOD.*Ncf1^*m1J*^*	**0.05**	NA	0.33	**0.04**
3	NOD.*Rag1^−/−^*.*Ncf1^*m*1*J*^*	NOD	NOD	**0.03**	0.33	NA	0.09
4	NOD.*Rag1^−/−^*.*Ncf1^*m*1*J*^*	NOD	NOD.*Ncf1^*m1J*^*	**0.01**	**0.04**	0.09	NA

* *NA indicates not applicable*.

§ *P-values in bold text are significant (p < 0.05)*.

### Maturation of NOD-*Ncf1^*m1J*^* and NOD DC Is Comparable

**Figure 2:**
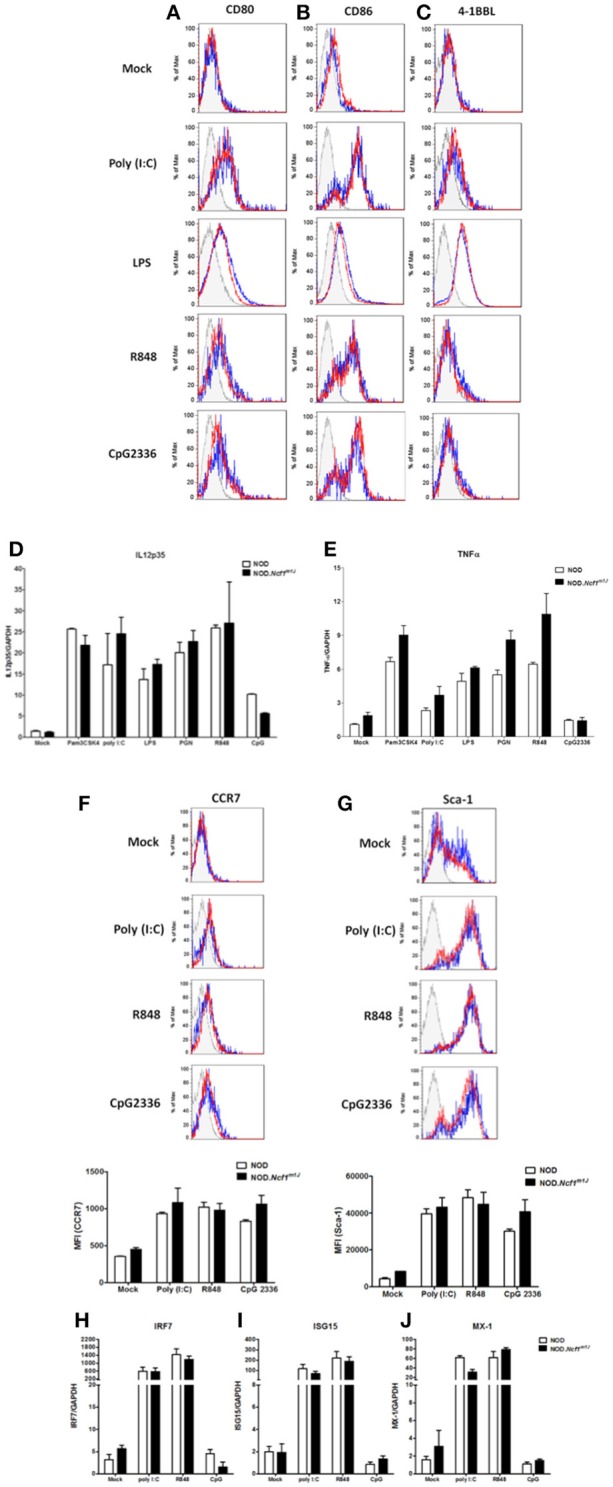
Maturation of DC from NOD and NOD-*Ncf1*^*m1J*^ mice with TLR ligands results in comparable upregulation of surface co-stimulatory molecules, cytokine transcription, and Type 1 interferon response genes. Histogram surface staining of BMDC from NOD and NOD-*Ncf1*^*m1J*^ for **(A)** CD80, **(B)** CD86, and **(C)** 4-1BBL after 24 h incubation with media alone (Mock), 25 μg/mL poly (I: C), 100 ng/mL LPS, 1 μg/mL R848, or 5 μg/mL CpG2336. Real-time quantitative PCR was performed for the target genes **(D)** IL12p35 and **(E)** TNFα in DCs after 12 h stimulation with: media alone (Mock) 100 ng/mL Pam3CSK4, 10 μg/mL PGN, 25 μg/mL poly (I: C), 100 ng/mL LPS, 1 ug/mL R848, or 5 μg/mL CpG2336. PCR were performed with pooled BMDC from three mice and results were compiled from three independent experiments done in triplicate. Histogram and the quantitation of **(F)** CCR7 and **(G)** Sca-1 after a 24 h stimulation of BMDC from NOD or NOD-*Ncf1*^*m1J*^ with 25 μg/mL poly (I: C), 1 μg/mL R848, and 5 μg/mL CpG2336. Histograms are representative. Each bar graph is compiled data from three independent experiments performed in triplicate. Real-time quantitative PCR of the type 1 interferon responsive genes **(H)** IRF7, **(I)** ISG15, and **(J)** MX-1 in NOD and NOD-*Ncf1*^*m1J*^ DCs after 24 h exposure to the indicated TLR ligand. PCR were performed with pooled BMDC from three mice and results were compiled from three independent experiments done in triplicate.

Maturation of DCs is one of the earliest events in the initiation of T1D (2, 37). In the process of maturation, DCs up-regulate surface co-stimulatory markers and MHC molecules in conjunction with the production of pro-inflammatory cytokines to prime antigen specific T cells. DCs can be activated by an array of Toll-Like Receptor (TLR) or Pattern Recognition Receptor (PRR) signaling pathways and in doing so, respond to infection and damage. Accordingly, many of the TLR signaling pathways have been reported to be associated with T1D (38, 39). To test if is required in TLR signal transduction and the ensuing up-regulation of co-stimulatory molecules on DC, bone marrow-derived DCs (BMDC) from NOD or NOD-*Ncf1*^*m1J*^ were treated for 24 h with either Poly(I:C), LPS, R848, or CpG2336 to activate TLR3, TLR4, TLR7/8, or TLR9, respectively. The activation markers CD80, CD86, and 4-1BBL were measured by surface staining and analyzed by multicolor flow cytometry (Figures 2A–C). All four ligands significantly boosted the expression of CD80 and CD86 (Figures 2A,B), while 4-1BBL was up-regulated by Poly(I:C) and LPS but not R848 nor CpG2336 (Figure 2C). When comparing BMDC from NOD and NOD-*Ncf1*^*m1J*^, DCs from both strains showed indistinguishable increases in surface expression (Figures 2A–C) suggesting the p47^phox^ was not required for co-stimulatory molecule up-regulation during DC maturation.

T_H_1 responses are the dominant path for pathogenic T cell differentiation in T1D. As professional APCs, DCs control CD4^+^ T cell differentiation though mechanisms of antigen presentation and co-stimulation as well as secretion of cytokines and ROS, which provide the “third signal” for activation (40, 41). IL-12 is the primary cytokine produced by DCs to drive the T_H_1 response (42, 43), and IL-12 is required for the priming of diabetogenic CD8^+^ T cells. We have previously demonstrated that macrophages from NOD-*Ncf*^*m1J*^ mice have compromised IL-12 production (24). Similarly, in the context of the B6 or B10 genetic background, Ncf1 has been proposed to play a suppressive role on IL-12 p35 production (44, 45). To investigate the role of Ncf1 in IL-12 expression by DCs and the impact on NOD T cell differentiation, IL-12p35 mRNA (Figure 2D) and production of the pro-inflammatory cytokine TNFα (Figure 2E) were examined 12 h after DC activation with the ligands for TLR1/2, TLR2/6, TLR4, TLR7, or TLR9, Activation of the series of TLRs significantly increased the level of IL-12p35 and TNFα mRNA. Interestingly, we found BMDCs from NOD-*Ncf*^*m1J*^ showed no significant deficiency in the transcription of these cytokines (Figures 2D,E). Overall, DC maturation in NOD-*Ncf*^*m1J*^ mice did not appear deficient in terms of the up-regulation of co-stimulatory molecules or pro-inflammatory cytokines upon targeted stimulations when compared to DCs from NADPH oxidase 2-intact NOD mice.

### Lack of Ncf1 Does Not Impact Production of Type 1 Interferons by DC

Type 1 interferons (T1-IFN) are required for the initiation of T1D (46). These cytokines also promote DC cross-presentation by modulating antigen survival within the phagosome, endocytic routing and processing, and hence enhance the cross-priming of islet specific CTLs (47, 48). To determine if Ncf1 regulates the expression and responses of T1-IFN by DCs, BMDC from NOD and NOD-*Ncf*^*m1J*^ were activated by Poly(I:C), R848, or CpG2336, and cell surface levels of CCR7 (Figure 2F) and stem cell antigen-1 (Sca-1, Figure 2G) were assessed by flow cytometry. CCR7 is a T1-IFN-induced chemokine receptor that functions to recruit DCs from peripheral tissue into lymphoid organs (49, 50). Sca-1 is a phosphatidylinositol-linked membrane protein that is also T1-IFN-induced on DCs. After 24 h of stimulation with TLR ligands, CCR7 was mildly upregulated (Figure 2F), while Sca-1 was significantly increased (Figure 2G) to an equal extent on DCs from both the NOD and NOD-*Ncf*^*m1J*^ strains.

To confirm that Ncf1 in DCs is not necessary for production and response of T1-IFN, transcription of the T1-IFN-inducible genes interferon regulatory factor-7 (IRF7), interferon stimulated gene 15 (ISG15), and myxoma response protein-1 (Mx1) in BMDCs from NOD or NOD-*Ncf1*^*m*1*J*^ were measured after 12-h stimulation by Poly(I:C), R848, or CpG2336 for respective activation of TLR3, TLR7/8, or TLR9. Significant elevations in the expression of IRF7 (Figure 2H), ISG15 (Figure 2I), and Mx1 (Figure 2J) were seen after stimulation with Poly(I:C) or R848, while small increases in transcription were observed following TLR9 activation with CpG2336 (Figures 2H,J). However, no differences were observed in NOD-*Ncf1*^*m1J*^ vs. NADPH oxidase 2 expressing DCs in terms of response to T1-IFN (Figures 2F–J). Taken together, Ncf1 in DCs does not play a role in the inducible T1-IFN production or response after activation of an array of TLRs.

### Comparable DC Phagocytosis in NOD and NOD-*Ncf^*m1J*^*

**Figure 3:**
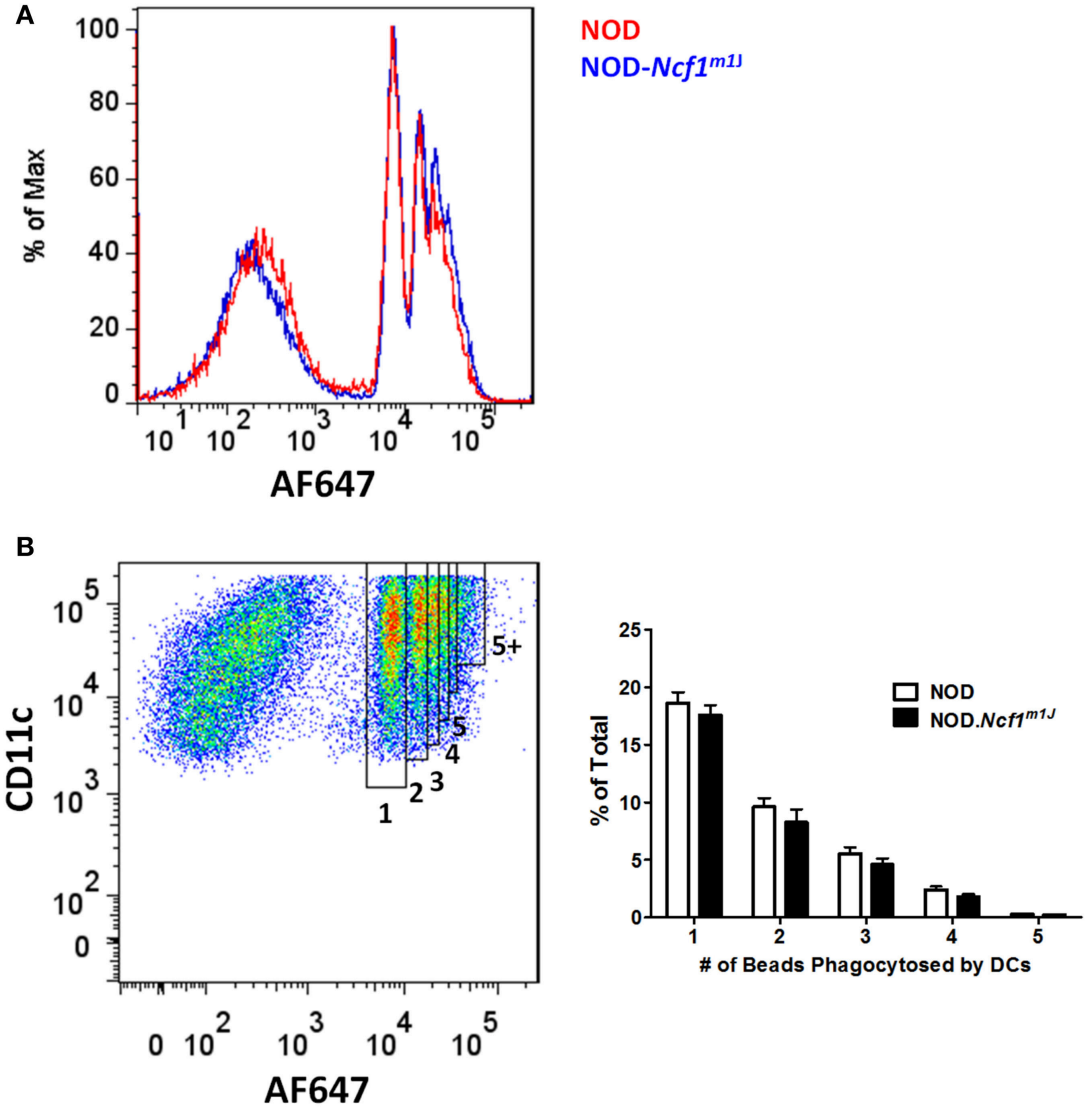
Normal antigen uptake in NOD-*Ncf1*^*m1J*^ DCs. **(A)** Representative histogram of DCs phagocytized different numbers beads. DCs from NOD or NOD-*Ncf1*^*m1J*^ were incubated with AF647 conjugated latex beads for 15 min and cells were analyzed by FACS after 3 washes. **(B)** Gating strategy and quantitation of DC phagocytosis. Each bar graph is representative of three independent experiments performed in triplicates.

Since Ncf1 is not required for DC maturation measured by upregulation of surface markers and production of pro-inflammatory cytokines following stimulation with various TLRs, we asked if ROS is necessary for the presentation of antigen by DCs. It has been reported that ROS produced by NADPH oxidase 2 is critical for the cross-presentation of antigen in MHC Class I by DCs to CD8^+^ T cells. Cross-presentation can be roughly divided into four steps: (1) phagocytosis of antigens; (2) limiting antigen degradation; (3) translocation of exogenous antigen into cytoplasm; and (4) presentation of antigens onto MHC Class I molecules. The proposed mechanism whereby NADPH oxidase 2 promotes an increase in antigen cross-presentation is through attenuating the phagosomal acidification following phagocytosis, thus facilitating antigen translocation into the cytoplasm (15, 21, 22). To examine if ROS is required for DC phagocytosis, we employed AlexaFluor 647 (AF647) labeled latex beads as a probe. Antibody-labeled (α-CD11c) BMDC from NOD and NOD-*Ncf*^*m1J*^ were incubated with AF647-labeled beads for 15 min at 37°C and subjected to flow cytometry (Figure 3A). Quantitative analysis of the CD11c positive population was conducted by gating on DCs that had taken up different numbers of beads. The percentage of DCs having phagocytized from one to five beads showed a declining trend (Figure 3B). However, bead uptake was comparable between NOD and NOD-*Ncf1*^*m1J*^ (Figure 3B). Thus, we concluded that Ncf1 is dispensable for antigen uptake by DCs.

### Enhanced Phagosomal Acidification in *NOD-Ncf1^*m1J*^* DCs

**Figure 4:**
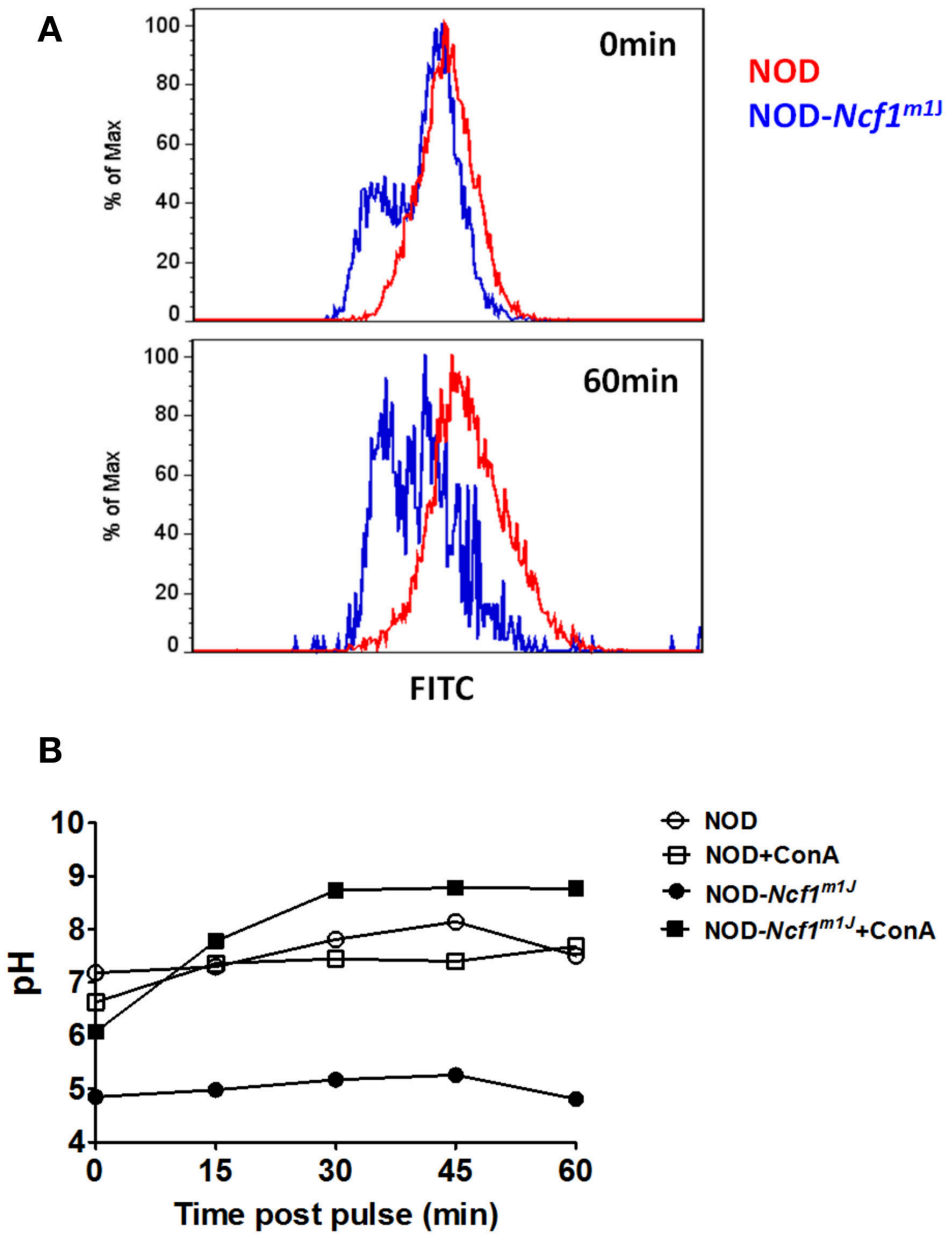
Enhanced phagosomal acidification in NOD-*Ncf1*^*m1J*^ DCs. **(A)** Representative histogram of DCs phagocytized FITC/AF647 linked beads. DCs from NOD or NOD-*Ncf1*^*m1J*^ were incubated with fluorescence linked latex beads for 15 min, then unbound beads were washed away. Cells were cultured 0, 15, 30, 45, or 60 additional minutes then cells were analyzed by FACS. **(B)** Phagosomal pH kinetics of DC from NOD or NOD-*Ncf1*^*m1J*^ post phagocytosis. DC were treated with or without 30 μg/mL V-ATPase inhibition with concanamycin A.

Although it has been reported that ROS are necessary for regulation of phagosomal acidification, some studies have argued that ROS are dispensable in phagosomal pH regulation (15, 17). These previous conflicting studies were conducted using the C57BL/6 (B6) background rather than the autoimmune-prone NOD mouse. One significant difference regarding antigen presentation between NOD and B6 mice involves the *Slc11a1* gene within the *Idd5* interval on Chromosome 1. Nramp1, the gene product of *Slc11a1*, promotes acidification of phagosomes in DCs and this protein is deficient in B6 mice (51) leading to compromised phagosomal acidification following phagocytosis. Here we used cells derived from NOD mice with a functional Nramp1 (52). To examine phagosomal acidification we utilized FITC, a widely used pH sensitive fluorescence dye. FITC conjugated ovalbumin (DQ-OVA) and the pH insensitive dye AF647 were linked to latex beads. BMDC were then incubated with these FITC/AF647-fluorescent beads. Notably, even with functional *Slc11a1* alleles, phagosomal pH in NOD DC was almost neutral through the initial 60 min after phagocytosis. In stark contrast, we observed rapid and significant decreases in phagosomal pH for *NOD-Ncf1*^*m1J*^ DC. Indeed, pH quickly dropped to around 5 within the first 15 min of the observation period (Figure 4), suggesting a rapid DC phagosomal acidification when Ncf1 was non-functional. The final pH value measured in NOD-*Ncf1*^*m1J*^ DCs was 1 unit lower than the previously reported values using gp91^phox^-defective B6 DCs (15), which could be rescued to approximately pH 7 by adding the V-ATPase inhibitor concanamycin A (Figure 4B). Overall, Ncf1 indispensable for NOD DCs to maintain a neutral pH shortly after phagocytosis.

### Accelerated Antigen Degradation in NOD-*Ncf1^*m1J*^* DCs

**Figure 5:**
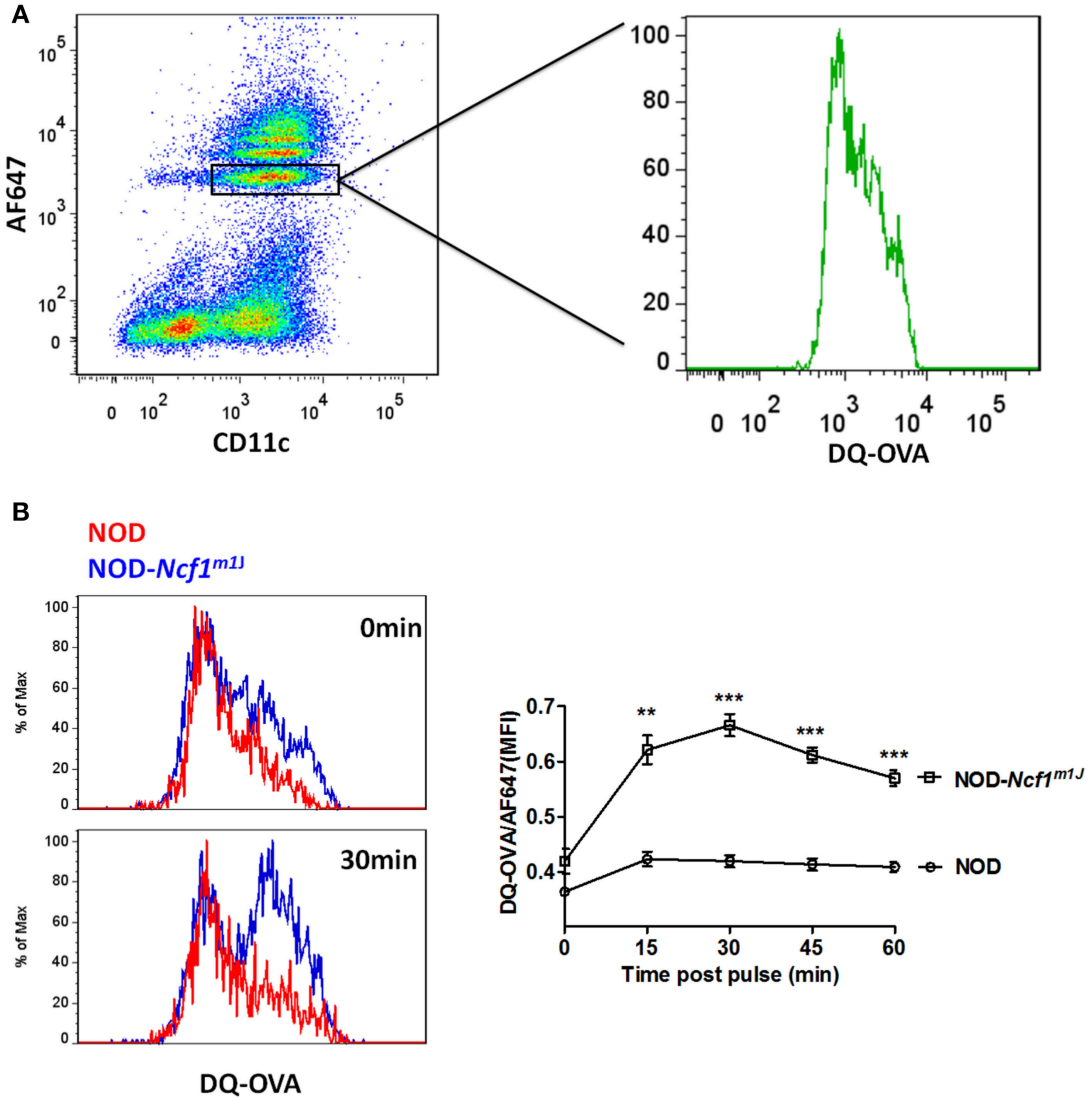
Accelerated antigen degradation in NOD-*Ncf1*^*m1J*^ DCs. **(A)** Illustration of DQ-OVA analysis using FACS. DCs were incubated with DQ-OVA/AF647 linked latex beads for 15 min and cells were analyzed by FACS at different time points after bead wash-off. **(B)** Histograms and quantitative analysis of DQ-OVA degradation. Data in the bar graph are represented as mean ± SEM. Statistical analysis used Student's *t*-test. ^**^p < 0.01; ^***^p < 0.001.

In DCs, for efficient cross-presentation of exogenous antigen, protein degradation is restricted immediately following phagocytosis. As shown in the previous reports and above, DCs constrain phagosomal acidification to inhibit antigen degradation (15, 21, 22). Due to the strong decrease of phagosome pH values observed in NOD-*Ncf1*^*m1J*^ DC, we expected to observe accelerated antigen digestion. To test this, we employed DQ-conjugated ovalbumin (DQ-OVA), which is designed to have self-quenched fluorescence group pairs when the three-dimensional conformation is intact. Upon degradation of this protein, the fluorescence and quenching groups detach from one another, and green fluorescence is emitted upon excitation (Figure 5A). Here, latex beads were linked with DQ-OVA and AF647 and incubated with BMDCs from NOD or NOD-*Ncf1*^*m1J*^ mice. DCs were analyzed, as described in the Materials and Methods by flow cytometry at the indicated time points post-phagocytosis. In NOD DCs, only a mild increase in fluorescence (~5%) was observed as late as 60 min after pulsing (Figure 5). This lack of antigen degradation is associated with maintenance of neutral pH (Figure 4). However, in NOD-*Ncf1*^*m1J*^ DC there was a rapid increase in FITC fluorescence intensity. Indeed, only 15 min post-pulsing, the fluorescence intensity of DQ-OVA increased by 50% (Figure 5B). Therefore, NOD-*Ncf1*^*m1J*^ DC rapidly exhibited a reduction in pH (Figure 4) and degradation of ovalbumin (Figure 5) indicating that without Ncf1, there is accelerated antigen degradation in the phagosome.

### Impaired Antigen Translocation in NOD-*Ncf1^*m1J*^* DC

**Figure 6:**
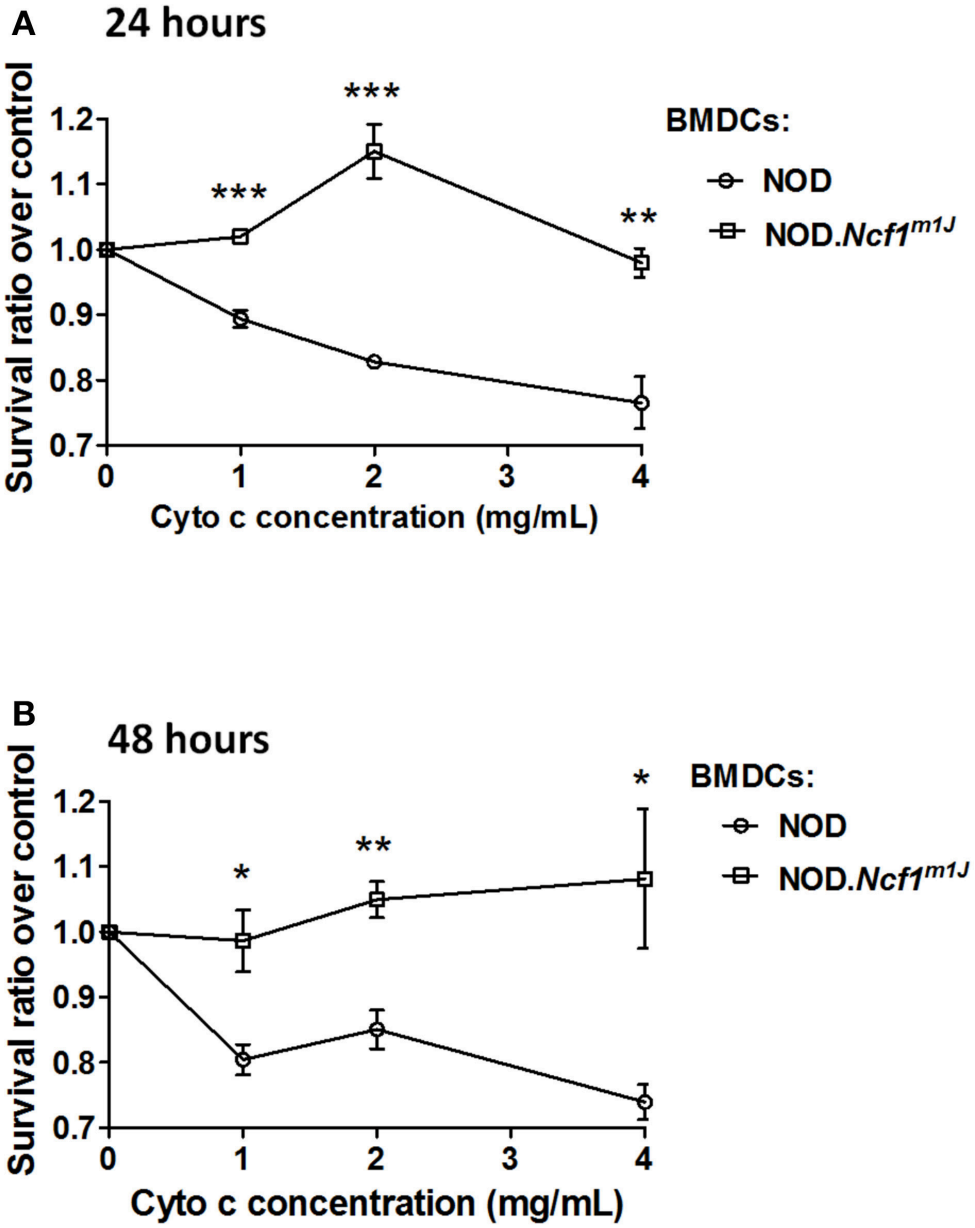
Deficient antigen translocation into cytoplasm in NOD-*Ncf1*^*m*1*J*^ DCs. DCs were treated with cytochrome c with various concentrations for 24 h **(A)** or 48 h **(B)**. Cell survival rate was represented as the ratio between treated and non-treated groups. Data are represented as mean ± SEM. Statistical analysis used Student's *t*-test. ^*^p < 0.05; ^**^p < 0.01; ^***^p < 0.001.

In cross-presentation, exogenous protein is exported into the cytoplasm by an unknown mechanism and processed by the proteasome for peptide antigen loading into MHC Class I molecules (14). To measure protein translocation into the cytosol, we employed cytochrome c as a probe. Cytochrome c is a protein that is sequestered in the inner mitochondrial membrane and functions as part of the electron transport chain. During apoptosis, cytochrome c is released into the cytoplasm and complexes into the apoptosome to induce apoptotic cell death. When cross-presenting cells such as BMDCs are incubated with recombinant cytochrome c, they will internalize cytochrome c and transport the intact protein from endosomes into cytoplasm, which triggers apoptosome activation and caspase-3 dependent apoptosis; in contrast, in cells that are incapable of translocating exogenous cytochrome c to cytoplasm, apoptosis would not be triggered (33, 34). Thus, we employed cellular apoptosis to indicate antigen translocation by incubating BMDC with cytochrome c. NOD and NOD-*Ncf1*^*m1J*^ DC were treated with increasing concentrations of cytochrome c [1 to 4 mg/mL] for 24 or 48 h, and apoptosis was measured by MTT assay. In both 24 and 48-h groups, cytochrome c induced a dose dependent killing of NOD DCs (Figure 6). However, cytochrome c did not induce apoptosis of NOD-*Ncf1*^*m1J*^ DCs indicating that cytochrome c was not transported into the cytoplasm in p47^phox^ deficient DCs (Figure 6), consistent with accelerated antigen degradation in NOD-*Ncf1*^*m1J*^ DC.

### Defective Cross Presentation in NOD-*Ncf1^*m1J*^* DC

**Figure 7:**
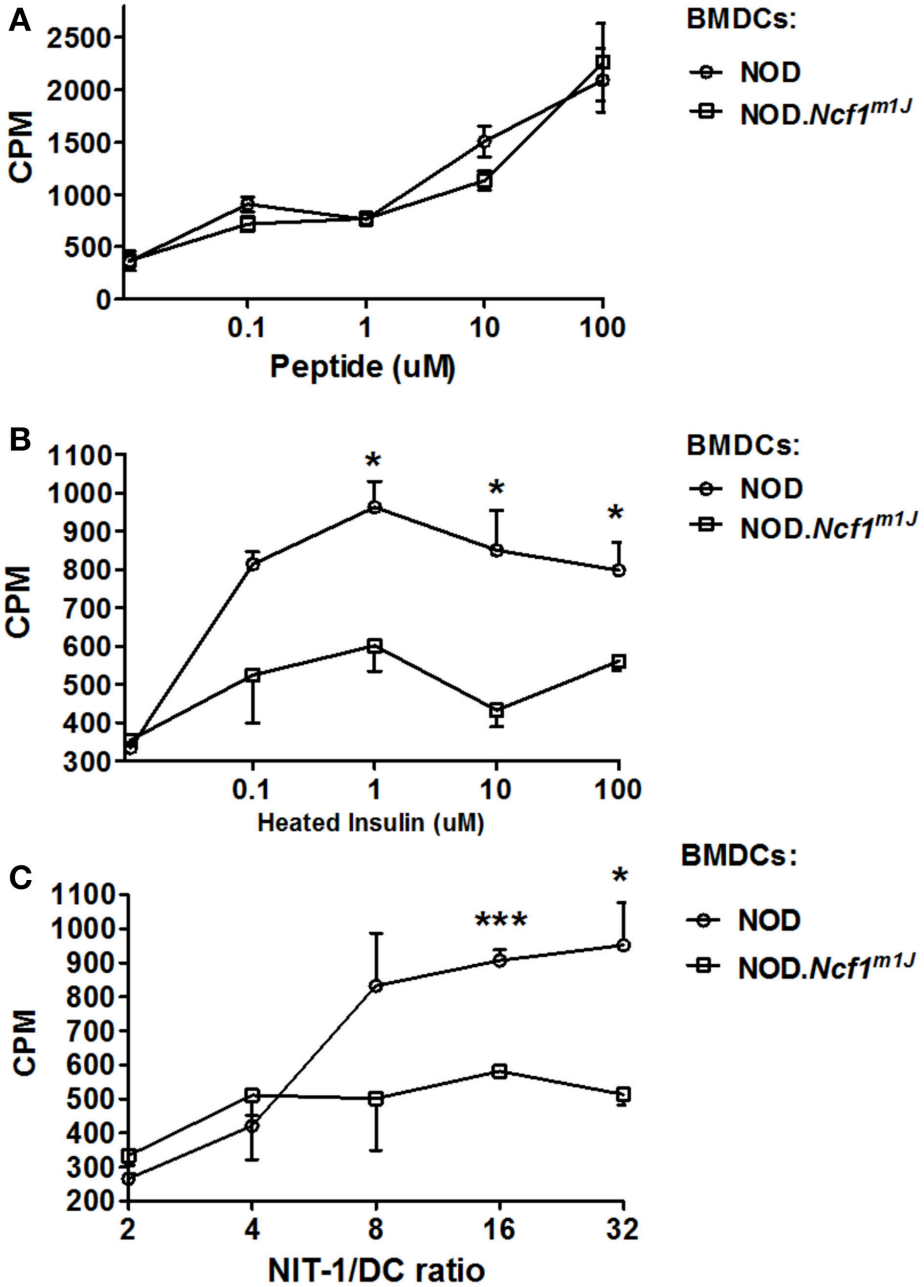
Cross-priming of autoreactive CD8^+^ T cells by DCs. **(A)** Proliferation of G9C8 cells induced by BMDCs from NOD or NOD-*Ncf1*^*m1J*^ pulsed with insulin peptide B15-23. **(B)** Proliferation of G9C8 cells induced by BMDCs from NOD or NOD-*Ncf1*^*m1J*^ pulsed with heat-inactivated insulin. **(C)** Proliferation of G9C8 cells induced by necrotic NIT-1 cell pulsed BMDCs. Graphs are representative of three independent experiments performed in triplicate. Data are represented as mean ± SEM. Statistical analysis used Student's *t*-test. ^*^p < 0.05; ^***^p < 0.001.

To determine if excessive antigen degradation and inhibition of antigen translocation have an impact on CTL activation, an *in vitro* cross-presentation assay was performed. BMDC from NOD-*Ncf1*^*m1J*^ or NOD were pulsed with antigen and incubated with G9C8 cells, an insulin specific CD8^+^ T cell clone (53). T cell proliferation was then measured by [^3^H]TdR incorporation. When DCs were pulsed with the specific insulin B15-23 peptide antigen, T cell proliferation was comparable in both groups. This suggests that when antigen processing is not required, NOD-*Ncf1*^*m1J*^ DCs have no deficiency in priming diabetogenic CD8^+^ T cells, consistent with the data above showing equal upregulation of co-stimulatory markers and pro-inflammatory cytokine expression by *Ncf1*-intact and -deficient DCs (Figure 2). In contrast, when DCs were primed with heat-inactivated insulin, G9C8 CD8^+^ T cell proliferation did not occur when the DCs were from NOD-*Ncf1*^*m1J*^ mice (Figure 7B). Strong G9C8 proliferation was induced when *Ncf1*-intact NOD DCs processed and presented heat-inactivated insulin (Figure 7B). Together, these data indicate that antigen cross-presentation is severely affected by the absence of Ncf1.

To mimic the scenario in T1D, necrotic NIT-1 cells (pancreatic β cell line derived from a NOD mouse) were added to DC-G9C8 co-cultures. As expected, NOD-*Ncf1*^*m1J*^ DCs were incapable of inducing diabetogenic T cell activation compared with the *Ncf1*-intact control DC (Figure 7C). This highlights the indispensable role of NADPH oxidase 2 in the DC to activate diabetogenic T cells in T1D. In summary, processing of exogenous autoantigen by NOD-*Ncf1*^*m1J*^ DCs is deficient, and without a functional Ncf1, DCs are not able to cross-present autoantigens to diabetogenic CD8^+^ T cells and drive the activation of these CTL.

## Discussion

ROS have long been considered a key part of T1D pathogenesis. While much effort has gone into identifying the cellular sources and targets of ROS, the overall roles for these molecules in T1D remain controversial. Due to the exquisite sensitivity of rodent islets to free radicals and oxidants, ROS have been proposed as a major mediator of β cell destruction. In addition, ROS are utilized by the immune system as signaling mediators. We have addressed the role of NADPH oxidase 2-produced ROS in both the islet and immune system. Our data support that NADPH oxidase 2-derived free radicals and oxidants are contributors to immunological events in T1D but have little responsibility in the islet (23, 54).

The role of NADPH oxidase 2 in β cell function and dysfunction has been recently clarified. While early reports noted that NADPH oxidase 2 was essential for both glucose-induced insulin secretion and pro-inflammatory cytokine mediated β cell destruction, more recent animal model and clinical studies have not confirmed these results (23, 54, 55). Pre-clinical studies have noted that NADPH oxidase 2 deficient mouse models carrying non-functional allele of either *Cybb* (gp91^phox^) or *Ncf1* are resistant to diabetes development and exhibit enhanced insulin secretion in response to glucose (23, 24, 54, 55). Further, our clinical studies have reported that humans with Chronic Granulomatous Disease (CGD), due to mutations in the gp91^phox^ subunit and no residual NADPH oxidase 2 activity, have reduced incidence of diabetes as compared to that in the general population (56). However, islets from NOD and NOD.*Ncf1*^*m1J*^ mice were destroyed at comparable levels when exposed to proinflammatory cytokines or pre-activated insulin-specific AI4 CD8^+^ T cells (23), thus suggesting that NADPH oxidase 2 deficient β cells are not resistant to autoimmune destruction. Indeed, adoptive transfer of pre-activated insulin-reactive AI4 CTLs rapidly induced T1D in both NOD and NOD-*Ncf1*^*m1J*^ mice (23). In stark contrast, transfer of naïve T cells into NOD-*Ncf1*^*m1J*^ mice resulted in delayed and reduced T1D onset compared to NADPH oxidase 2 intact mice [Figure 1 and (23)]. The dichotomy of these results supports an important role of NADPH oxidase 2 activity in the CTL axis rather than in β cells.

Our studies have noted that the almost complete T1D resistance of NOD-*Ncf1*^*m1J*^ mice was associated with T cell signaling and differentiation that was skewed away from a diabetogenic T_H_1 response and resulted in defective effector function (24). T_H_1 responses could be, in part, rescued by interactions with NADPH oxidase 2 intact APCs suggesting that during T cell activation by APC, ROS signals in the immune synapse regulate T cell differentiation (24). Further studies demonstrated that CD8^+^ T cells from NOD-*Ncf1*^*m1J*^ mice exhibited defective activation and effector function. When transferred into *Ncf1*-intact NOD mice, NOD-*Ncf1*^*m1J*^ CD8^+^ T cells initiated T1D with significantly slower kinetics when compared to the transfer of CD8^+^ T cells from NOD mice (23). This defect resulted from an inability of NOD-*Ncf1*^*m1J*^ CTL to properly deactivate the redox-sensitive signaling suppressor Tumor Suppressor Complex 1/2 (TSC1/2). After activation of NOD-*Ncf1*^*m1J*^ CTL, TSC1/2 remained active and continued to suppress mTORC1. This suppression resulted in a lack of T-bet transcriptional activity along with reduced expression of IFNγ and granzyme B. The limited expression of these effector molecules impaired CTL effector function and inhibited *in vitro* β cell cytotoxicity. Therefore, oxidants generated by NADPH oxidase are essential to deactivate TSC1/2 during T cell activation. However, the defects in CD8^+^ T cell activity are not enough to explain the almost full T1D resistance of NOD-*Ncf1*^*m1J*^ mice. Our adoptive transfer studies clearly demonstrate that CD8^+^ T cells from NOD-*Ncf1*^*m1J*^ mice exhibit increased pathogenesis when transferred into NOD.*Rag1*^−/−^ mice in comparison to transfer into intact NOD-*Rag1*^−/−^*Ncf1*^*m1J*^ mice (Figure 1) leading to our prediction that additional non-T cell mechanisms are involved.

Herein, we assessed the competence of Ncf1-deficient DCs for activation of diabetogenic CD8^+^ T cells via cross-presentation of exogenous protein antigens. We observed that NOD-*Ncf1*^*m1J*^ DC were deficient in their ability to stimulate autoreactive CD8^+^ T cells after phagocytosis of intact antigen or necrotic β cells. These data suggest that the reduction in T1D observed in NOD-*Ncf1*^*m1J*^, results, in part, from a defect in the ability of NOD-*Ncf1*^*m1J*^ DCs to activate autoreactive T cells (23). The dearth of DC-induced autoreactive CD8^+^ T cell activation did not result from compromised expression of co-stimulatory molecules or pro-inflammatory cytokines (Figure 2). In fact, under stimulation conditions with a panel of TLR ligands, the co-stimulatory molecules CD80, CD86, and 4-BBL were upregulated similarly when comparing NOD and NOD-*Ncf1*^*m1J*^ DCs (Figures 2A–C). T1-IFN production and responses were also intact in NOD-*Ncf1*^*m1J*^ DCs after addition of ligands for TLR3, TLR7, or TLR9 (Figures 2F–J). These data suggest that on the NOD background ROS is dispensable for the activation of MyD88 as well as TRIF after TLR ligand binding. The findings are consistent with the data showing that when NOD-*Ncf1*^*m1J*^ DC were pulsed with the specific peptide antigen, autoreactive CTLs were activated (Figure 7). Taken together, these data demonstrate that NOD-*Ncf1*^*m1J*^ DCs can efficiently prime diabetogenic CD8^+^ T cells when antigen processing is not required, and thereby, highlight the potential significance of NADPH oxidase 2 in processing of autoantigens for presentation by MHC Class I. In addition, these data provide support for ROS in regulating T1D through antigen-cross presentation by DC, a concept has not previously been proposed or discussed.

While roles for NADPH oxidase 2 in antigen cross-presentation by DCs have been previously reported, a controversy remains regarding the mechanism of action for NADPH oxidase 2 in this process (15, 17). Consistent with previous reports (15, 21, 22), our data support the postulate that antigen uptake is not impaired in NOD.*Ncf1*^*m1J*^ DC, suggesting that NADPH oxidase 2 is dispensable for phagocytosis (Figure 3). However, when measuring antigen degradation and phagosomal pH in DC, we observed a rapid degradation of antigen and a swift decline in pH values by DC that lacked a functional ph47^phox^ subunit (Figures 4–6). In accord with previous work (15), this acidification as well as rapid antigen degradation could be rescued by inhibition of the V-ATPase with concanamycin A. At the concentration used, concanamycin A is highly specific for V-ATPases and can be used to probe biological mechanisms of V-ATPase mediated acidification (15, 57). In the presence of concanamycin A, phagosome pH was strongly increased in DC from *NOD-Ncf1*^*m1J*^ mice (Figure 4B). As the pH remained neutral within phagosomes of NOD DCs (with a functional Ncf1) after phagocytosis of antigen, only a mild pH increase was observed during concanamycin A treatment (Figure 4B). These results are in agreement with the previous demonstration that V-ATPase is the major regulator of endosomal acidification that can be regulated by NADPH oxidase 2 (15).

These findings support the model where NADPH oxidase 2 modulates antigen processing though prevention of phagosomal acidification (15, 22). In addition, as NOD mice have an intact *Slc11a1* (52), increased phagosomal acidification would be expected as compared to B6 mouse models utilized in prior studies (15, 17, 22). Consistently, we observed a larger pH drop in p47^phox^ deficient NOD DCs compared with the reported results using DC from B6-*Cybb*^−/−^ mice (15). Due to the acidic environment in the phagosome, the antigen degradation is strongly accelerated in NOD-*Ncf1*^*m1J*^ DCs, which results in the deficiency in the antigen translocation from phagosome into cytoplasm (Figure 6). The combination of NRAMP1 activity with a loss of NADPH oxidase 2 function results in decreased antigen loading in MHC Class I and reduced DC cross-presentation of autoantigens in T1D. This is confirmed by the inability of NOD-*Ncf1*^*m1J*^ DC to activate insulin-reactive CD8^+^ T cells when provided heat-inactivated insulin or necrotic β cells as the antigen source (Figure 7). Our study supports the hypothesis that ROS promote cross presentation by regulating phagosomal pH values and confirm that NADPH oxidase 2-derived ROS from diabetogenic DCs are required to activate autoreactive CD8^+^ T cells through promoting the preservation of autoantigens for MHC Class I cross-presentation.

## Ethics Statement

All studies herein were approved by the institutional animal care and use committee (IACUC) at the University of Florida (UF) and performed under UF-IACUC Protocol #201605475.

## Author Contributions

CL researched the data and wrote the manuscript, RW, AL, YX, and JC researched the data and reviewed/edited the manuscript. AS, MW, and JL contributed to discussion and reviewed/edited the manuscript, and CM conceived of the study, researched the data, and wrote/reviewed/edited the manuscript.

### Conflict of Interest Statement

The authors declare that the research was conducted in the absence of any commercial or financial relationships that could be construed as a potential conflict of interest.

## Abbreviations

AF647: Alexa Fluor 647
APC: Allophycocyanin
APC: Antigen presenting cells
BMDC: Bone Marrow Dendritic Cells
CGD: Chronic Granulomatous Disease
CTL: Cytotoxic CD8^+^ T cell
DC: Dendritic cells
DQ-OVA: DQ-conjugated ovalbumin
FITC: Fluorescein isothiocyanate
NGZB: Granzyme B
IFNγ: Interferon gamma
IRF7: Interferon regulatory factor-7
ISG15: Interferon stimulated gene 15
LPS: Lipopolysachharide
Mx1: Myxoma response protein-1
NOD: Non-obese diabetic
OVA: Ovalbumin
Pam3CSK4: Pam3CysSerLys4
PLN: Pancreatic draining lymph nodes
PRR: Pattern Recognition Receptor
PE: Phycoerythrin
Poly(I:C): Polyinosinic-polycytidylic acid
ROS: Reactive oxygen species
rmGM-CSF: Recombinant mouse granulocyte-macrophage colony stimulating factor
Sca-1: Stem cell antigen-1
TLR: Toll-Like Receptor
T1D: Type 1 Diabetes
T1-IFN: Type 1 interferon
TSC1/2: Tumor Suppressor Complex 1/2.
